# Expandable Cage Versus Mesh Cage for the Treatment of Vertebral Osteomyelitis

**DOI:** 10.7759/cureus.100922

**Published:** 2026-01-06

**Authors:** Mitsuhiro Nishizawa, Mladen Djurasovic, Steven D Glassman, John R Dimar, Charles H Crawford, Benjamin A Kostic, Leah Y Carreon

**Affiliations:** 1 Orthopedic Surgery, University of Louisville School of Medicine, Louisville, USA; 2 Orthopedics, Norton Leatherman Spine Center, Norton Healthcare, Louisville, USA; 3 Research, Norton Leatherman Spine Center, Norton Healthcare, Louisville, USA

**Keywords:** anterior debridement, cage, corpectomy, spine infection, vertebral osteomyelitis

## Abstract

Background and objective

Consensus regarding the optimal method for anterior column reconstruction in vertebral osteomyelitis has not been established. Direct comparisons between mesh and expandable cages have not been reported. This study aimed to evaluate differences in clinical outcomes, including revision rates, recurrence rates, complications, and costs, between these two reconstruction methods, as well as to identify independent risk factors for treatment failure in patients undergoing anterior column reconstruction with metal implants.

Methods

Clinical records of consecutive patients who underwent surgical intervention for vertebral osteomyelitis at a single institution between 2012 and April 2024 were reviewed. Patients who underwent anterior column debridement with either a mesh or an expandable cage, along with posterior fusion involving four or more levels, were included. Clinical outcomes, including nonunion, revisions, recurrence, and costs, were compared between the mesh and expandable cage groups.

Results

Ninety-two patients were included: 20 (22%) in the expandable cage group and 72 (78%) in the mesh cage group. The expandable cage group had a significantly higher nonunion rate (35% vs. 9.7%, p = 0.014) and revision rate (30% vs. 9.7%, p = 0.032). Costs were also higher in the expandable cage group, with a significant difference in in-hospital costs (Expandable: $77,737 ± 34,586; Mesh: $63,250 ± 27,899; p = 0.028). Multivariable analysis identified the number of fused levels (OR = 1.32, 95% CI: 1.05-1.71; p = 0.024) and the use of an expandable cage (OR = 3.27, 95% CI: 1.04-11.02; p = 0.046) as independent risk factors for treatment failure, defined as revision surgery, recurrence, or mortality.

Conclusions

This study is the first to compare outcomes between expandable cages and mesh cages in patients with vertebral osteomyelitis. The findings suggest that the use of an expandable cage for reconstruction after anterior column debridement is associated with higher costs and increased risks of nonunion and revision surgery compared with mesh cages. Additionally, the use of an expandable cage was identified as an independent risk factor for treatment failure in vertebral osteomyelitis.

## Introduction

Vertebral osteomyelitis is a serious infection associated with substantial morbidity and mortality, and its incidence has increased over the past several decades [1,2]. Although antibiotic therapy remains the mainstay of treatment, surgical intervention is indicated in cases of antibiotic failure, sepsis, neurological deficits, or mechanical instability [3,4]. Thorough removal of infected tissue is essential for successful treatment [5]. However, anterior column reconstruction is often challenging due to large bone defects resulting from infection and debridement [6].

Several options exist for anterior column reconstruction in patients with vertebral osteomyelitis. Bone grafting with autografts has traditionally been considered the gold standard; however, it is increasingly avoided due to donor site morbidity, the risk of complications, and the need for an additional surgical procedure [7,8]. As an alternative, titanium mesh cages are widely used for anterior column reconstruction and have been reported to yield favorable outcomes, including superior mechanical stability and a lower recurrence rate, despite some criticism regarding their use in the setting of active infection [9,10]. Additionally, several studies have demonstrated positive outcomes with expandable titanium cages, although their high cost is a notable drawback [11,12].

Despite these advances, consensus regarding the optimal method for anterior column reconstruction in vertebral osteomyelitis has not been established, and no study has directly compared mesh cages with expandable cages. This study aims to evaluate differences in clinical outcomes, including revision rates, recurrence rates, complications, and costs, between these two reconstruction methods. Furthermore, we aim to identify independent risk factors for treatment failure in patients undergoing anterior column reconstruction with metal implants.

## Materials and methods

We retrospectively reviewed the clinical records of consecutive patients who underwent surgical intervention for vertebral osteomyelitis at a single institution between 2012 and April 2024. All patients were evaluated by an infectious disease specialist to determine appropriate antibiotic therapy, which typically consisted of a six-week course of intravenous antibiotics. Surgical intervention was indicated for patients with sepsis, persistent or uncontrolled infection despite antibiotic therapy, neurological deficits, or spinal instability.

Patients were included if they underwent anterior column debridement with placement of either a titanium expandable cage or a titanium mesh cage, along with posterior fusion involving four or more levels, and had available follow-up data at six months. Patients who underwent shorter fusions were excluded to reduce variability in surgical complexity and ensure comparability of outcomes among those with large bony defects requiring extensive reconstruction. Patients with a history of spine surgery within the previous 90 days who subsequently developed an infection at or near the surgical site, or whose disposition was unknown, were also excluded.

Patient demographics, including age, sex, BMI, American Society of Anesthesiologists (ASA) physical status classification, smoking status, and comorbidities, were collected. The location of vertebral osteomyelitis and clinical presentation, including sepsis, neurological deficits, epidural abscess, and concomitant infections, were documented. Causative pathogens were identified through blood cultures or intraoperative specimens. The interval from diagnosis to surgery was recorded. Surgical data, including the number of fusion levels, staged procedures, vertebral body replacement, estimated blood loss (EBL), operative time, and cage height, were also documented. In addition, the ratio of cage height to vertebral body height (cage-to-body ratio) was calculated.

Patients were categorized into two groups based on the type of cage used: expandable cage (Expandable) or titanium mesh cage (Mesh). All cages were filled with morselized healthy autograft, allograft, demineralized bone matrix, or recombinant human bone morphogenetic protein and inserted into the bone defect after thorough debridement of infected or necrotic tissue. The choice of cage and bone graft material was determined by the operating surgeon based on intraoperative assessment. Pre- and postoperative antibiotic regimens were managed by an infectious disease specialist. Preoperative regimens were empiric, providing coverage for both Gram-negative and Gram-positive organisms, or tailored based on blood culture results. Postoperative regimens were guided by intraoperative Gram stain and culture findings.

Hospital length of stay, fusion status, revision surgery, recurrence of vertebral osteomyelitis, 30-day and one-year postoperative complications, and 30-day and one-year readmissions were assessed. Fusion was evaluated using plain radiographs or CT and was defined as the presence of bridging bone or a substantial fusion mass surrounding the cages at the last follow-up. Postoperative complications included implant-related complications (e.g., rod or screw fracture and cage subsidence), surgical complications (e.g., surgical site infection, wound dehiscence, and epidural hematoma), and major medical complications (e.g., cardiovascular events, respiratory failure, pneumonia, deep vein thrombosis or pulmonary embolism, acute kidney injury, gastrointestinal events, urinary tract infection, Clostridium difficile infection, and death). Treatment failure was defined as revision surgery, recurrence of infection, or death [13,14].

Costs incurred for surgery, in-hospital stay, and postoperative care from discharge to 90 days, as well as total costs from admission to 90 days, were provided by a senior financial analyst.

Statistical analysis

Continuous variables were summarized as mean ± SD, and categorical variables as frequencies and percentages. The Shapiro-Wilk test was used to assess the normality of continuous variables. Student’s t-test was applied to compare normally distributed continuous variables between groups, while the Mann-Whitney U test was used for non-normally distributed continuous variables. Categorical variables were compared using Fisher’s exact test.

As a subgroup analysis, factors associated with treatment failure were evaluated using a multiple logistic regression model. In addition to age and sex, variables with a p-value <0.1 in the univariable analysis were included in the multivariable model. Statistical significance was defined as a p-value <0.05. All analyses were performed using R software (version 4.4.2).

This study was reviewed and approved by the University of Louisville Institutional Review Board and was determined to be exempt under 45 CFR 46.101(b), Category 4: secondary research for which consent is not required, involving the use of identifiable private information or identifiable biospecimens.

## Results

Of the 270 patients screened, 92 met the inclusion criteria and were included in the analysis. The mean age of the cohort was 56.8 ± 13.0 years, and 64% were male. The mean follow-up duration was 23.9 ± 22.7 months. Among the 92 patients, 20 (22%) received expandable cages (Expandable group), while 72 (78%) received mesh cages (Mesh group). The mean cage height and cage-to-body ratio were 44.6 ± 16.8 mm and 174.1 ± 57.5% in the Expandable group and 36.4 ± 17.0 mm and 155.2 ± 85.5% in the Mesh group, respectively, with no significant differences between groups (p = 0.062 and p = 0.145, respectively).

There were no significant differences between groups in patient demographics, clinical presentation, or most surgical data, except for the incidence of methicillin-resistant *Staphylococcus aureus *(MRSA) (Expandable: 12, 60% vs. Mesh: 23, 32%, p = 0.036) and EBL (Expandable: 746.5 ± 369.1 mL vs. Mesh: 1075.9 ± 663.1 mL, p = 0.020) (Table 1).

**Table 1:** Comparison of patient demographics, clinical presentation, and surgical data between patients with expandable cage (Expandable) and mesh cage (Mesh) ᵃ p-Value from unpaired t-test ᵇ p-Value from Fisher’s exact test ᶜ p-Value from Mann-Whitney U test p < 0.05 was considered statistically significant. ASA, American Society of Anesthesiologists; CNS, coagulase-negative staphylococci; EBL, estimated blood loss; MRSA, methicillin-resistant *Staphylococcus aureus*; MSSA, methicillin-sensitive *Staphylococcus aureus*

Variable	Overall	Expandable	Mesh	p-Value
No. of patients, N	92	20	72	-
Age, years, mean (SD)	56.8 (13.0)	54.0 (15.4)	57.6 (12.2)	0.361ᵃ
Male sex, N (%)	59 (64%)	12 (60%)	47 (65%)	0.793ᵇ
BMI, kg/m², mean (SD)	28.5 (6.5)	27.5 (7.9)	28.8 (6.0)	0.363ᵃ
ASA grade, mean (SD)	3.2 (0.6)	3.3 (0.6)	3.2 (0.6)	0.936ᵇ
Current smoker, N (%)	40 (43%)	12 (60%)	28 (39%)	0.126ᵇ
Systemic steroid use, N (%)	6 (6.5%)	0 (0%)	6 (8.3%)	0.333ᵇ
Diabetes mellitus, N (%)	38 (41%)	9 (45%)	29 (40%)	0.799ᵇ
Intravenous drug use, N (%)	32 (35%)	7 (35%)	25 (35%)	1.000ᵇ
Clinical presentation, N (%)				
Sepsis	33 (36%)	9 (45%)	24 (33%)	0.430ᵇ
Neurological deficit	22 (24%)	6 (30%)	16 (22%)	0.555ᵇ
Epidural abscess	46 (50%)	14 (70%)	32 (44%)	0.075ᵇ
Concomitant infection	39 (42%)	11 (55%)	28 (39%)	0.213ᵇ
Identified organisms, N (%)				
MSSA	13 (14%)	3 (15%)	10 (14%)	1.000ᵇ
MRSA	35 (38%)	12 (60%)	23 (32%)	0.036ᵇ
CNS	8 (8.7%)	3 (15%)	5 (6.9%)	0.365ᵇ
Streptococcus	7 (7.6%)	1 (5.0%)	6 (8.3%)	1.000ᵇ
Escherichia coli	5 (5.4%)	2 (10%)	3 (4.2%)	0.297ᵇ
Serratia	5 (5.4%)	0 (0%)	5 (6.9%)	0.389ᵇ
Location, N (%)				
Cervical	12 (13%)	4 (20%)	8 (11%)	0.285ᵇ
Thoracic	41 (45%)	9 (45%)	32 (44%)	1.000ᵇ
Lumbar	39 (42%)	7 (35%)	32 (44%)	0.610ᵇ
Surgical variables				
No. of fusion levels, mean (SD)	6.1 (2.0)	6.6 (2.2)	5.9 (1.9)	0.167ᶜ
Staged surgery, N (%)	83 (90%)	16 (80%)	67 (93%)	0.099ᵇ
Vertebrectomy, N (%)	50 (54%)	13 (65%)	37 (51%)	0.319ᵇ
Operative time, min, mean (SD)	332.8 (102.3)	318.7 (97.8)	336.7 (103.9)	0.623ᵇ
EBL, mL, mean (SD)	1,004.3 (624.7)	746.5 (369.1)	1,075.9 (663.1)	0.020ᶜ
Cage height, mm, mean (SD)	38.2 (17.2)	44.6 (16.8)	36.4 (17.0)	0.062ᶜ
Cage-to-body ratio, %, mean (SD)	159.3 (80.4)	174.1 (57.5)	155.2 (85.5)	0.145ᶜ
Transfusion, N (%)	66 (72%)	13 (65%)	53 (74%)	0.575ᵇ
Diagnosis to surgery, days, mean (SD)	23.0 (37.2)	25.1 (35.7)	22.4 (37.8)	0.949ᶜ
Follow-up, months, mean (SD)	23.9 (22.7)	16.0 (13.7)	26.1 (24.3)	0.081ᶜ

Compared with patients in the Mesh group, those in the Expandable group had a significantly lower rate of solid fusion at the last follow-up (Expandable: 8, 40%; Mesh: 49, 68%; p = 0.036) and a significantly higher rate of nonunion (Expandable: 7, 35%; Mesh: 7, 9.7%; p = 0.014) (Table 2). The revision rate was also significantly higher in the Expandable group than in the Mesh group (Expandable: 6, 30%; Mesh: 7, 9.7%; p = 0.032). Although the recurrence rate of osteomyelitis was higher in the Expandable group (Expandable: 5, 25%; Mesh: 6, 8.3%), the difference did not reach statistical significance (p = 0.057). No significant differences were observed between the groups in other complications, readmissions, or mortality.

**Table 2:** Comparison of outcomes and complications between patients with an expandable cage (Expandable) and a mesh cage (Mesh) ᵇ p-Value from Fisher’s exact test ᶜ p-Value from Mann-Whitney U test p < 0.05 was considered statistically significant.

Variable	Overall	Expandable	Mesh	p-Value
No. of patients, N	92	20	72	-
Fusion status, N (%)				
Solid fusion	57 (62%)	8 (40%)	49 (68%)	0.036ᵇ
Indeterminate	13 (14%)	2 (10%)	11 (15%)	0.726ᵇ
Nonunion	14 (15%)	7 (35%)	7 (9.7%)	0.014ᵇ
Implant-related complications, N (%)				
Rod or screw fracture	7 (7.6%)	1 (5.0%)	6 (8.3%)	1.000ᵇ
Cage subsidence	16 (18%)	4 (20%)	12 (17%)	0.746ᵇ
Revision surgery, N (%)	13 (18%)	6 (30%)	7 (9.7%)	0.032ᵇ
Nonunion	6 (6.5%)	3 (15%)	3 (4.2%)	0.114ᵇ
Implant failure	5 (5.4%)	2 (10%)	3 (4.2%)	0.297ᵇ
Adjacent segment disease	6 (6.5%)	1 (5.0%)	5 (6.9%)	1.000ᵇ
Time to revision, months, mean (SD)	19.0 (15.1)	12.0 (11.3)	23.4 (16.2)	0.212ᶜ
Surgical complications, N (%)				
Surgical site infection	6 (6.5%)	0 (0%)	6 (8.3%)	0.333ᵇ
Wound dehiscence	9 (9.8%)	0 (0%)	9 (13%)	0.197ᵇ
Epidural hematoma	1 (1.1%)	1 (5.0%)	0 (0%)	0.217ᵇ
Recurrence, N (%)	11 (12%)	5 (25%)	6 (8.3%)	0.057ᵇ
Length of hospitalization, days, mean (SD)	16.7 (9.5)	17.3 (7.6)	16.6 (10.1)	0.509ᶜ
Major medical complications, N (%)				
90 days	71 (76%)	14 (70%)	57 (79%)	0.383ᵇ
1 year	76 (83%)	15 (75%)	61 (85%)	0.327ᵇ
Readmission, N (%)				
90 days	27 (34%)	5 (25%)	26 (36%)	0.430ᵇ
1 year	49 (53%)	8 (40%)	41 (57%)	0.211ᵇ
Death, N (%)	16 (17%)	5 (25%)	11 (15%)	0.327ᵇ

The costs were higher in the Expandable group than in the Mesh group (Table 3), with significant differences in cage cost (Expandable: US$5,464 ± 3,089; Mesh: US$783 ± 1,364; p < 0.01), surgery cost (Expandable: US$36,393 ± 19,960; Mesh: US$24,715 ± 8,185; p < 0.001), and in-hospital cost (Expandable: US$77,737 ± 34,586; Mesh: US$63,250 ± 27,899; p = 0.028).

**Table 3:** Comparison of costs between patients with expandable cages (Expandable) and mesh cages (Mesh), reported in United States dollars, mean (SD) ᵃ p-Value from unpaired t-test p < 0.05 was considered statistically significant.

Category	Expandable	Mesh	p-Valueᵃ
Cage, USD	5,464 (3,089)	783 (1,364)	<0.001
Surgery, USD	36,393 (19,960)	24,715 (8,185)	<0.001
In-hospital, USD	77,737 (34,586)	63,250 (27,899)	0.028
Discharge to 90 days, USD	5,374 (9,959)	4,796 (17,149)	0.443
Total cost from admission to 90 days, USD	83,111 (37,629)	68,046 (40,138)	0.069

In a subgroup analysis, 35 patients (38%) were classified as treatment failure cases. Based on the univariable analysis, age, sex, number of fused levels, staged surgery, and use of an expandable cage were included in the multivariable logistic regression model (Table 4).

**Table 4:** Comparison of patient demographics and surgical data between patients with successful treatment and those with treatment failure (revision surgery, recurrence, or one-year mortality) ᵃ p-Value from unpaired t-test ᵇ p-Value from Fisher’s exact test ᶜ p-Value from Mann-Whitney U test p < 0.05 was considered statistically significant. ASA, American Society of Anesthesiologists; CNS, coagulase-negative staphylococci; EBL, estimated blood loss; MRSA, methicillin-resistant *Staphylococcus aureus*; MSSA, methicillin-sensitive *Staphylococcus aureus*

Variable	Successful	Failure	p-value
No. of patients, N	57	35	-
Age, years, Mean (SD)	55.1 (13.1)	59.5 (12.5)	0.182ᵃ
Male, N (%)	36 (63%)	23 (66%)	0.827ᵇ
BMI, kg/m², Mean (SD)	28.4 (6.5)	28.7 (6.5)	0.806ᵃ
ASA grade, Mean (SD)	3.2 (0.6)	3.3 (0.6)	0.317ᵇ
Current smoker, N (%)	23 (40%)	17 (49%)	0.518ᵇ
Systemic steroid, N (%)	2 (3.5%)	4 (11%)	0.196ᵇ
Diabetic, N (%)	27 (47%)	11 (31%)	0.190ᵇ
Intravenous drug use, N (%)	22 (39%)	10 (29%)	0.373ᵇ
Clinical presentation, N (%)			
Sepsis	17 (30%)	16 (46%)	0.179ᵇ
Nerve deficit	11 (19%)	11 (31%)	0.214ᵇ
Epidural abscess	28 (49%)	18 (51%)	1.000ᵇ
Concomitant infection	24 (42%)	15 (43%)	1.000ᵇ
Identified organisms, N (%)			
MSSA	8 (14%)	5 (14%)	1.000ᵇ
MRSA	21 (37%)	14 (40%)	0.827ᵇ
CNS	5 (8.8%)	3 (8.6%)	1.000ᵇ
Streptococcus	4 (7.0%)	3 (8.6%)	1.000ᵇ
Escherichia coli	4 (7.0%)	1 (2.9%)	0.827ᵇ
Serratia	3 (5.3%)	2 (5.7%)	1.000ᵇ
Location, N (%)			
Cervical	5 (8.8%)	7 (20%)	0.200ᵇ
Thoracic	25 (44%)	16 (46%)	1.000ᵇ
Lumbar	27 (47%)	12 (34%)	0.279ᵇ
Surgery			
No. of fusion levels, Mean (SD)	5.6 (1.7)	6.7 (2.3)	0.030ᶜ
Staged surgery, N (%)	54 (95%)	29 (83%)	0.079ᵇ
Vertebrectomy, N (%)	27 (47%)	23 (66%)	0.131ᵇ
Operative time, min, Mean (SD)	332.8 (111.1)	332.7 (87.5)	0.541ᶜ
EBL, mL, Mean (SD)	1,003.7 (574.7)	1,005.3 (707.3)	0.800ᶜ
Cage height, mm, Mean (SD)	37.9 (18.3)	38.6 (15.5)	0.576ᶜ
Cage-to-body ratio, %, Mean (SD)	154.3 (81.0)	167.5 (79.8)	0.325ᶜ
Expandable cage, N (%)	8 (14%)	12 (34%)	0.036ᵇ
Transfusion, N (%)	41 (72%)	25 (71%)	1.000ᵇ
Diagnosis to surgery, days, Mean (SD)	21.0 (37.4)	26.2 (37.2)	0.199ᶜ

Treatment failure was independently associated with the number of fused levels (OR = 1.32, 95% CI: 1.05-1.71; p = 0.024) and the use of an expandable cage (OR = 3.27, 95% CI: 1.04-11.02; p = 0.046) (Table 5).

**Table 5:** Multiple logistic regression analysis of treatment failure (revision surgery, recurrence, or one-year mortality)

Variable	OR (95% CI)	p-Value
Age, years	1.04 (0.99-1.08)	0.06
Sex (Male)	1.06 (0.41-2.83)	0.906
No. of fusion levels	1.32 (1.05-1.71)	0.024
Staged surgery (Yes/No)	0.27 (0.04-1.41)	0.126
Expandable cage (Yes/No)	3.27 (1.04-11.02)	0.046

## Discussion

This study is the first to compare outcomes between expandable cages and mesh cages in patients with vertebral osteomyelitis. The findings suggest that the use of an expandable cage for reconstruction after anterior column debridement is associated with higher costs and increased risks of nonunion and revision surgery compared with mesh cages. Additionally, the use of an expandable cage was identified as an independent risk factor for treatment failure in vertebral osteomyelitis.

Expandable cages are a valuable option for anterior column reconstruction, offering several advantages over mesh cages, particularly in cases of vertebral osteomyelitis in which soft tissue and vascular involvement limit access to the vertebral body [11,15]. Compared with mesh cages, expandable cages require less surgical exposure and can be more easily inserted into the bone defect after corpectomy through a smaller surgical corridor, resulting in a lower risk of vascular injury and reduced EBL, as demonstrated in this study [16]. In addition, their expandable design allows height restoration according to the size of the defect and enables greater kyphosis correction [17].

Although limited in number, previous reports on the use of expandable cages in vertebral osteomyelitis have shown relatively favorable outcomes comparable to those reported for autografts [18,19]. Lu et al. reported a 6% recurrence rate and no implant failures in 36 patients [19]. Robinson et al. reported no cases of nonunion, implant failure, or recurrence in 25 patients [20]. Klute et al. reported no recurrences and a 20% revision rate in 24 patients [21]. Neuhoff et al. reported a 9% recurrence rate and an 11% implant failure rate in 100 patients [22].

However, in the present study, although there were no differences in cage height or cage-to-body ratio, which reflect the size of the bone defect after debridement, the use of an expandable cage was associated with a greater economic burden and significantly higher rates of nonunion (35%) and revision surgery (30%) compared with mesh cages. These findings are consistent with a systematic review and meta-analysis of expandable cages used in cervical corpectomy for patients with cervical spondylosis, which reported a higher risk of nonunion and reoperation [23,24]. These complications have been attributed to factors such as the limited contact surface area and the minimal space available for bone graft material in expandable cages compared with mesh cages [25-27].

Our study also identified the use of an expandable cage as an independent risk factor for treatment failure, including revision surgery, recurrence, or mortality. The higher prevalence of MRSA infection in the expandable cage group may have contributed to this finding, as MRSA is a known risk factor for recurrence and persistent infection, which can lead to nonunion or mortality [28,29]. Therefore, the observed differences in outcomes between groups may be partially confounded by pathogen-related factors rather than cage type alone.

Furthermore, the use of an expandable cage was associated with increased economic burden. The costs of the cage, surgery, and in-hospital care were significantly higher in the expandable group. Although no significant differences were observed in postoperative costs after discharge, the cost differential was likely underestimated, as the analysis included data only up to 90 days after surgery. Additional revision surgeries, which were more frequent in the expandable group, typically occur beyond this period and would further increase the total cost associated with expandable cages.

This study has several limitations. First, it was a retrospective analysis conducted at a single institution. Second, as expected in this patient population, a substantial number of patients were lost to follow-up, resulting in a relatively small sample size. Third, segmental angle and spinal alignment were not assessed. Finally, the decision to use an expandable or mesh cage was not randomized but determined by the surgeon, and thus, the potential for selection bias cannot be excluded. Despite these limitations, this is the first study to directly compare mesh cages and expandable cages in the treatment of vertebral osteomyelitis. It provides meaningful insight into surgical outcomes and complications associated with expandable cages and helps address an important gap in the current literature. Further large-scale, prospective, randomized controlled trials are warranted to determine the optimal implant choice for the surgical management of vertebral osteomyelitis.

## Conclusions

In this study comparing outcomes between expandable cages and mesh cages in patients with vertebral osteomyelitis, the use of an expandable cage for reconstruction after anterior column debridement was associated with higher costs and increased risks of nonunion and revision surgery compared with mesh cages. The use of an expandable cage was identified as an independent risk factor for treatment failure in vertebral osteomyelitis.
